# Efficacy of the association liver partition and portal vein ligation for staged hepatectomy for the treatment of solitary huge hepatocellular carcinoma: a retrospective single-center study

**DOI:** 10.1186/s12957-021-02199-1

**Published:** 2021-03-30

**Authors:** Zhenfeng Deng, Zongrui Jin, Yonghui Qin, Mingqi Wei, Jilong Wang, Tingting Lu, Ling Zhang, Jingjing Zeng, Li Bao, Ya Guo, Minhao Peng, Banghao Xu, Zhang Wen

**Affiliations:** 1 Department of Hepatobiliary Surgery, The First Affiliated Hospital of Guangxi Medical University, Nanning, Guangxi China; 2 Department of Ultrasound, The First Affiliated Hospital of Guangxi Medical University, Nanning, Guangxi China; 3 Department of Radiology, The First Affiliated Hospital of Guangxi Medical University, Nanning, Guangxi China; 4 Department of Pathology, The First Affiliated Hospital of Guangxi Medical University, Nanning, China; 5 Key Laboratory of Cancer Prevention and Therapy, Tianjin Medical University Cancer Institute and Hospital, National Clinical Research Center for Cancer, Tianjin, China

**Keywords:** ALPPS, Solitary huge hepatocellular carcinoma, Future liver remnant, Outcomes

## Abstract

**Background:**

The feasibility of association liver partition and portal vein ligation for staged hepatectomy (ALPPS) for solitary huge hepatocellular carcinoma (HCC, maximal diameter ≥ 10 cm) remains uncertain. This study aims to evaluate the safety and the efficacy of ALPPS for patients with solitary huge HCC.

**Methods:**

Twenty patients with solitary huge HCC who received ALPPS during January 2017 and December 2019 were retrospectively analyzed. The oncological characteristics of contemporaneous patients who underwent one-stage resection and transcatheter arterial chemoembolization (TACE) were compared using propensity score matching (PSM).

**Results:**

All patients underwent complete two-staged ALPPS. The median future liver remnant from the ALPPS-I stage to the ALPPS-II stage increased by 64.5% (range = 22.3–221.9%) with a median interval of 18 days (range = 10–54 days). The 90-day mortality rate after the ALPPS-II stage was 5%. The 1- and 3-year overall survival (OS) rates were 70.0% and 57.4%, respectively, whereas the 1- and 3-year progression-free survival (PFS) rates were 60.0% and 43.0%, respectively. In the one-to-one PSM analysis, the long-term survival of patients who received ALPPS was significantly better than those who received TACE (OS, *P* = 0.007; PFS, *P* = 0.011) but comparable with those who underwent one-stage resection (OS, *P* = 0.463; PFS, *P* = 0.786).

**Conclusion:**

The surgical outcomes of ALPPS were superior to those of TACE and similar to those of one-stage resection. ALPPS is a safe and effective treatment strategy for patients with unresectable solitary huge HCC.

**Supplementary Information:**

The online version contains supplementary material available at 10.1186/s12957-021-02199-1.

## Background

Hepatocellular carcinoma (HCC) is a lethal malignancy with a poor prognosis and limited therapeutic options, accounting for approximately 841,000 new cases and 782,000 deaths worldwide each year [1]. Current treatment strategies for HCC mainly include surgical resection, transplantation, radiofrequency ablation, transarterial chemoembolization, chemotherapy, and radiotherapy [2]. Surgical resection allows patients to acquire better long-term survival than other treatments in most cases [3, 4] and remains the major treatment for HCC [2, 5]. Considering the radical surgical treatment for HCC, the extensive radical resection for tumor removal may lead to insufficient future liver remnant (FLR) and subsequently cause posthepatectomy liver failure and even death. A huge HCC (maximal diameter ≥ 10 cm) is a common and direct factor for the deficiency of FLR in clinical practice. Patients with huge HCC frequently lose the chance of radical treatment, thereby limiting the feasibility of the hepatic resection. Previous studies indicate that the 5-year overall survival (OS) in patients with huge HCC who have undergone nonsurgical treatment is less than 20%, which is significantly lower than those who have undergone surgical resection (about 25–40%) [6–8].

The association liver partition and portal vein ligation for staged hepatectomy (ALPPS), an innovative procedure of hepatectomy, can effectively induce the rapid hypertrophy of FLR within a short time [9–11]. This strategy of accelerated regeneration enables extensive hepatic resection well beyond the safe resection of 70% of liver volume [12], creating an opportunity for patients with unresectable HCC. Although ALPPS is controversial in the initial application due to severe complications and high early mortality, the situation has occurred in the evidently improved prognosis of ALPPS over time. A continuous decrease in the postoperative morbidity and the early mortality makes ALPPS reach standard outcomes accepted for major liver surgery [13]. ALPPS increases the resection rates of HCC. However, patients with solitary huge HCC who have undergone ALPPS may have increased potential risks because of the bulky tumor volume and the insufficient FLR. Currently, the efficacy and the safety of ALPPS for the treatment of solitary huge HCC are rarely reported and remain uncertain.

This study aims to explore the feasibility of ALPPS in the treatment of patients with solitary huge HCC. The propensity score matching (PSM) analysis is used, and the oncologic outcomes of one-stage resection for resectable solitary huge HCC are compared with those of the transcatheter arterial chemoembolization (TACE) for the unresectable tumor.

## Methods

### Study design and ethics

In this study, consecutive patients diagnosed with solitary huge HCC (maximal diameter ≥ 10 cm) and underwent ALPPS at the First Affiliated Hospital of Guangxi Medical University between January 2017 and December 2019 were enrolled. The oncological data of these patients were retrospectively analyzed and compared with those of patients who underwent one-stage resection and TACE during the same period by using the PSM analysis. This study was approved by the local Ethics Committee (approval number: 2020KY-E-110) and conducted in accordance with the 1990 Declaration of Helsinki and the following amendments. Written informed consents were obtained from each patient before using their clinical data for research.

### Patient selection

Transient elastography (TE) and imageological examination, such as computed tomographic (CT), magnetic resonance imaging (MRI), or ultrasonography, were combined to evaluate the preoperative degree of liver fibrosis or cirrhosis [14–16]. The FLR value and the standard liver volume (SLV) of each patient were calculated at the same time. The insufficient FLR was defined as follows: (1) FLR/SLV < 50%, severe fibrosis or cirrhosis; (2) FLR/SLV < 40%, mild/moderate fibrosis; and (3) FLR/SLV < 30%, without liver fibrosis or cirrhosis. In this study, patients with solitary huge HCC and sufficient FLR underwent one-stage resection, and patients with insufficient FLR underwent ALPPS or TACE. In addition, the choice of treatment strategy should be based on the patient’s intentions and preoperative liver function.

Patients receiving TACE were diagnosed with HCC by using two of three imageological examinations (i.e., CT, MRI, and ultrasonography) combined with the serum alpha-fetoprotein (AFP) level > 400 ng/mL or by needle biopsy to determine the suspected diagnosis. Finally, the maximum tumor diameter was measured using imageological examinations to diagnose the solitary huge HCC. All image analysis was interpreted by two or more professional reviewers with an inter-observer agreement.

Patients who had received any initial treatment for HCC, such as chemotherapy, radiotherapy, or sorafenib, within 6 months were excluded from this study. Patients with incomplete clinical records and lost to follow-up were excluded.

### Volumetric measurement

The FLR volume was measured using the digital software of intelligent/interactive qualitative and quantitative analyses (IQQA-Liver; EDDA Technology Inc., Princeton, NJ). The SLV was calculated on the basis of the estimation formula of standard liver volume for Chinese adults [17]. Thus, the preoperative FLR/SLV was measured to determine whether FLR was insufficient. The increases in the volume of FLR after the ALPPS-I stage were confirmed to assess whether to continue to the ALPPS-II stage. The following conditions were considered to proceed with the operation of the ALPPS-II stage [18]: (1) FLR/SLV ≥ 50% with severe fibrosis or cirrhosis, (2) FLR/SLV ≥ 40% with mild/moderate fibrosis, and (3) FLR/SLV ≥ 30% without liver fibrosis or cirrhosis.

### Surgical procedures

In the operation of the ALPPS-I stage, the abdomen was first opened through the reverse “L” incision and explored to confirm any distant metastasis in the peritoneum. The intraoperative ultrasonography was used to re-evaluate the location, size, number of tumor, and the position of adjacent blood vessels especially the anatomical positional relationship of the right, middle, and left hepatic veins. The liver resection line along or near the right side of the falciform ligament was subsequently marked, and the gallbladder was removed. The right portal vein was separated and ligated. Finally, the liver parenchyma was transected, and occlusion of the middle and left hepatic veins was performed to reduce intraoperative bleeding. Patients were confirmed without bile leakage before closing the abdomen.

The operation of the ALPPS-II stage was performed when the volumetric measurement demonstrated sufficient FLR volume and the patient’s overall condition was acceptable. In the operation of the ALPPS-II stage, the abdomen was opened again along the original surgical incision. The right hepatic artery, right portal vein, right hepatic duct, short hepatic vein, and perihepatic ligaments were transected to remove the tumor-bearing liver. Tumor specimens were used for pathological diagnosis.

### Follow-up

Patients were termly followed once every month from discharge to the first 3 months and every 3–6 months thereafter in our outpatient department of liver surgery. The main contents of the follow-up include imageological examinations, chest radiography, liver function, and serum AFP level.

The OS and the progression-free survival (PFS) rates of each patient after the PSM analysis were calculated until August 1, 2020. The survival time was defined as the period between therapeutic operations and the date of death or last contact. The terminal event of PFS included distant metastasis, recurrence, and death after ALPPS and one-stage resection, extrahepatic or intrahepatic metastasis, and death after TACE.

### PSM analysis

The PSM analysis was performed to minimize the effect of patient selection bias and baseline differences between patients who received ALPPS and one-stage resection or TACE. The 1:1 matching with a 0.1 caliper width was constructed using the logistic regression model on the basis of the age, gender, body mass index, model for end-stage liver disease (MELD) score, serum AFP level, Child-Pugh grade, tumor size, vascular invasion, and extrahepatic metastasis. The variable balance between the matched groups was assessed using the paired *t* test, the chi-square test, or the 2-tailed Fisher exact test. The OS and PFS of ALPPS were compared with one-stage resection and TACE after PSM analysis.

### Statistical analysis

Quantitative variables were expressed as median with ranges, and categorical variables were presented as the number of cases with percentage. The 1:1 matching between ALPPS and one-stage resection/TACE cohorts was performed using the PSM functional module in the SPSS [19]. The Kaplan-Meier method was used to calculate the 1- and 3-year OS and PFS rates. The log-rank test was used to assess the differences in survival outcomes. The Cox regression analysis was carried out to identify the potential risk factors for poor outcomes. All statistical analyses were performed using the SPSS software (version 20; IBM, Chicago, IL). *P* < 0.05 was considered statistically significant.

## Results

Twenty patients diagnosed with solitary huge HCC had undergone ALPPS at our hospital during January 2017 and December 2019. These patients had a single tumor in the liver with a median diameter of 14.5 cm (range = 10.0–21.0 cm). The median age was 47 years (range = 32–75 years). The median preoperative MELD score was 5 (range = 2–11), and the median rate of the indocyanine green retention at 15 min was 4.4% (range = 2.4–10.9%). In accordance with the Child-Pugh classification, 19 (95%) and 1 (5%) patients were classified as classes A and B, respectively, whereas in accordance with the Barcelona Clinic Liver Cancer staging, 7 (35%), 5 (25%), and 8 (40%) patients were classified as stages A, B, and C, respectively. The baseline characteristics at preoperation are shown in Table 1.

**Table 1:** Preoperative characteristics of patients who underwent ALPPS

Variable	ALPPS (*n*=20)
Age, years	47 (32~75)
Gender, female/male, *n* (%)	3 (15%)/17 (85%)
BMI, kg/m^2^	21.3 (18.0~30.1)
ECOG score, 0/1/2, *n* (%)	4 (20.0%)/13 (65.0%)/3 (15.0%)
AFP, ≥400ng/mL/<400ng/mL, *n* (%)	12 (60%)/8 (40%)
Charlson comorbidity index	4 (3~7)
TE for the degree of liver fibrosis, *n* (%)	
No fibrosis	2 (10%)
Mild fibrosis	1 (5%)
Moderate fibrosis	2 (10%)
fibrosis	3 (15%)
Cirrhosis(%)	12 (60%)
MELD score	5 (2~11)
ICGR_15_, %	4.4 (2.4~10.9)
Child-Pugh class, A/B/C, *n* (%)	19 (95%)/1 (5%)/0 (0%)
BCLC staging, A/B/C, *n* (%)	7 (35%)/5 (25%)/8 (40%)

*Abbreviations*: *ALPPS*, association liver partition and portal vein ligation for staged hepatectomy; *BMI*, body mass index, *ECOG*, Eastern Cooperative Oncology Group; *AFP*, alpha-fetoprotein; *TE*, transient elastography; *MELD*, model for end-stage liver disease; *ICGR*_*15*_, indocyanine green retention rate at 15 min; *BCLC*, Barcelona Clinic Liver Cancer

### Intraoperative and postoperative data of ALPPS

All patients received the complete two-staged ALPPS operation. A total of 17 (85%) patients underwent the right hemihepatectomy ALPPS, 2 (10%) patients underwent the extended right hemihepatectomy ALPPS, and 1 (5%) patient underwent the right trisectionectomy ALPPS. The median interval between the ALPPS-I and the ALPPS-II stages was 18 days (range = 10–54 days).

No anesthesia accident occurred during the ALPPS operation. Eighteen patients met the criteria for negative margins (R0 resection), and the two remaining patients underwent the R1 resection. Moreover, one patient had bile leakage after the operation of the ALPPS-II stage. The incidences of severe complications (classification ≥ III) were 20% and 25% after the ALPPS-I and the ALPPS-II stages, respectively. The postoperative pathological diagnosis verified that the pathological types of these 20 patients were all HCC. The intraoperative and the postoperative data are summarized in Table 2.

**Table 2:** Intra- and postoperative data of patients who underwent ALPPS

Variable	ALPPS-I stage	ALPPS-II stage
Operative time, min	364 (226~507)	337 (213~531)
Blood loss, mL	300 (100~2600)	775 (200~6000)
Blood transfusion, mL	300 (0~900)	250 (0~2150)
Postoperative bile leakage, *n* (%)		
No	20 (100%)	19 (95%)
Yes	0 (0%)	1 (5%)
Clavien-Dindo classification, *n* (%)		
I	11 (55%)	7 (35%)
II	5 (25%)	8 (40%)
III	2 (10%)	3 (15%)
IV	2 (10%)	2 (10%)
ISGLS classification, *n* (%)		
A	9 (45%)	3 (15%)
B	11 (55%)	16 (80%)
C	0 (0%)	1 (5%)
Ishak fibrosis score	/	3 (1~6)
Ishak inflammation score	/	5 (2~12)

*Abbreviations*: *ALPPS*, association liver partition and portal vein ligation for staged hepatectomy; *ISGLS*, International Study Group of Liver Surgery

### Volumetric assessment during ALPPS procedure

**Fig. 1:**
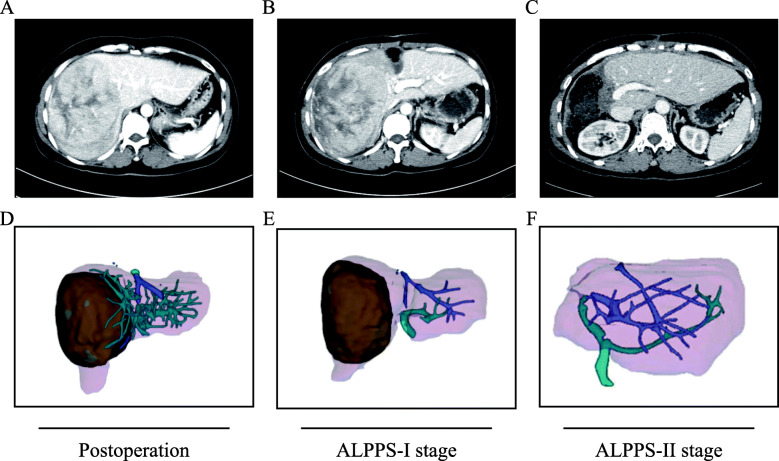
Examples of the CT diagram and the IQQA 3D reconstructed model of the liver in patients with solitary huge HCC during perioperation of ALPPS. Abbreviations: CT, computed tomographic; IQQA, intelligent/interactive qualitative and quantitative analyses

The CT diagram of the liver and the reconstructed 3D model via IQQA during perioperation of ALPPS are illustrated in Fig. 1. The liver volume-related data, including SLV, FLR, FLR/SLV ratio, and the increases in FLR, are presented in Table 3. The median increase in the FLR volume between the ALPPS-I and the ALPPS-II stages was 64.5% (range = 22.3–221.9%), and the FLR volume at preoperation of the ALPPS-II stage was significantly higher than that of the ALPPS-I stage (*P* < 0.001).

**Table 3:** Liver volume assessment during ALPPS procedure

Variable	
SLV, cm^3^	1013.0 (874.9~1231.6)
Preoperation of ALPPS-I stage	
FLR, cm^3^	388.8 (192.0~477.2)
FLR/SLV, %	36.6 (19.0~47.4)
Preoperation of ALPPS-II stage	
FLR, cm^3^	578.6 (402.1~823.0)
FLR/SLV, %	57.6 (43.7~85.1)
FLR increase between ALPPS-I and II stages, %	64.5 (22.3~221.9)
Absolute KGR, cm^3^/day	22.2 (13.1~42.3)
Relative KGR, %/day	2.1 (0.8~10.2)

*Abbreviations*: *ALPPS*, association liver partition and portal vein ligation for staged hepatectomy; *SLV*, standard liver volume; *FLR*, future liver remnant; *KGR*, kinetic growth rate

**Fig. 2:**
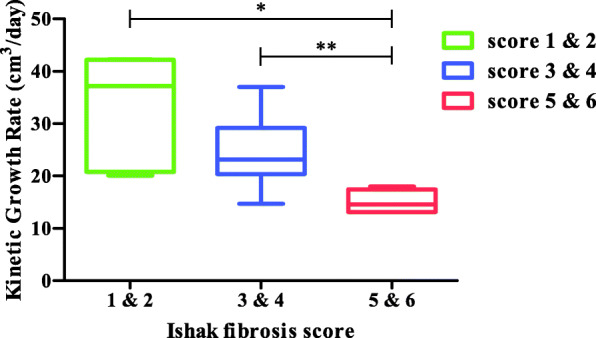
Relationship of Ishak fibrosis scores and KGR of FLR between the ALPPS-I and the ALPPS-II stages. The median KGR of patients with Ishak fibrosis scores of 1&2, 3&4, and 5&6 were 37.2, 23.2, and 14.6 cm^3^/day, respectively. The KGR values of patients with Ishak fibrosis scores of 1&2 or 3&4 were significantly higher than those with Ishak fibrosis scores of 5&6 (scores 1&2 vs. 5&6: *P* = 0.014; scores 3&4 vs. 5&6: *P* = 0.009). Abbreviations: KGR, kinetic growth rate; FLR, future liver remnant; ALPPS, association liver partition and portal vein ligation for staged hepatectomy

The absolute and the relative kinetic growth rates (KGR) of FLR were 21.7 cm^3^/day (range = 11.5–42.3 cm^3^/day) and 2.1%/day (range = 0.8–10.2%/day), respectively. The median KGR of patients with Ishak fibrosis scores of 1&2, 3&4, and 5&6 were 37.2 (range 20.1–42.3 cm^3^/day), 23.2 (range 14.7–37.0 cm^3^/day), and 14.6 (range 13.1–18.0 cm^3^/day) cm^3^/day, respectively (Fig. 2). Patients with Ishak fibrosis scores of 1&2 or 3&4 had significantly higher KGR than those with Ishak fibrosis scores of 5&6 (scores 1&2 vs. 5&6: *P* = 0.014; scores 3&4 vs. 5&6: *P* = 0.009).

### Survival analysis

The median follow-up time of all patients with solitary huge HCC who underwent ALPPS was 21.3 months (range = 12.1–41.6 months). One patient (5%) died within 90 days after surgery of the ALPPS-II stage. The 1- and 3-year OS rates of the ALPPS group were 70.0% and 57.4%, respectively. The 1- and 3-year PFS rates were 60.0% and 43.0%, respectively. The tumor recurrence, pulmonary metastasis, and death were observed in 1, 2, and 8 patients, respectively, during follow-up. The univariable Cox regression analysis identified the MELD score and the postoperative complications of the ALPPS-II stage ≥ III as risk factors for poor OS. Further multivariate analysis indicated that the two factors significantly affected the outcome after the ALPPS procedure (Table S1).

**Fig. 3:**
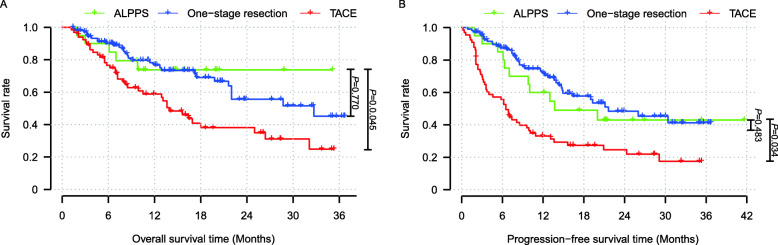
Survival analyses of total patients enrolled in this study. No significant difference was observed between ALPPS and one-stage resection patients on (**a**) the OS (*P* = 0.770) and (**b**) the PFS (*P* = 0.483) rates. However, (**a**) the OS (*P* = 0.045) and (**b**) the PFS (*P* = 0.034) rates of patients with ALPPS were significantly better than those with TACE. Abbreviations: ALPPS, association liver partition and portal vein ligation for staged hepatectomy; OS, overall survival; PFS, progression-free survival; TACE, transcatheter arterial chemoembolization

Survival analysis was performed to total patients enrolled in this study including 20 cases that underwent treatment for ALPPS, 110 for one-stage resection, and 66 for TACE. The results showed that the 1- and 3-year OS rates of all patients with one-stage resection were 76.7% and 45.2%, respectively (Fig. 3a, *P* = 0.770), and the 1- and 3-year PFS rates were 72.0% and 41.3%, respectively (Fig. 3b, *P* = 0.483), no significant difference compared with ALPPS in survival outcomes. In addition, the 1- and 3-year OS rates of all patients with TACE were 58.9% and 24.9%, respectively (Fig. 3a, *P* = 0.045), and the 1- and 3-year PFS rates were 33.1% and 17.5%, respectively (Fig. 3b, *P* = 0.034); patients with ALPPS had significantly better outcomes than those with TACE.

**Fig. 4:**
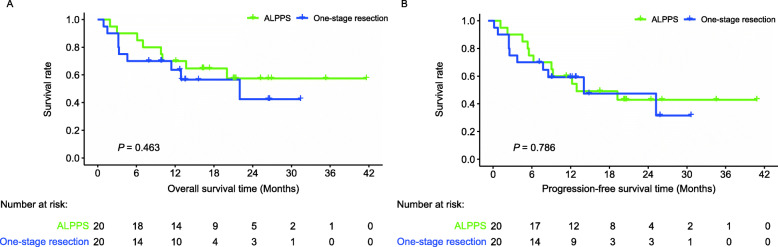
Survival analyses between ALPPS and the matched one-stage resection groups. No significant difference was observed between the ALPPS and the one-stage resection groups on (**a**) the OS (*P* = 0.463) and (**b**) the PFS (*P* = 0.786) rates. Abbreviation: ALPPS, association liver partition and portal vein ligation for staged hepatectomy; OS, overall survival; PFS, progression-free survival

To reduce the influence of confounding factors for survival analysis, PSM analysis was used to further compare the outcomes of different therapy strategies for patients with solitary huge HCC. During the same research period, 20 of 110 patients with solitary huge HCC who underwent one-stage resection were selected to match those who received ALPPS, which made the oncological characteristics between the two groups as close as possible by the PSM analysis (Table S2). The 1- and 3-year OS rates of the matched one-stage resection group were 63.6% and 42.4%, respectively (Fig. 4a, *P* = 0.463). The 1- and 3-year PFS rates were 59.2% and 31.6%, respectively (Fig. 4b, *P* = 0.786). The survival analysis revealed no significant difference in the OS and PFS of ALPPS and one-stage resection groups.

**Fig. 5:**
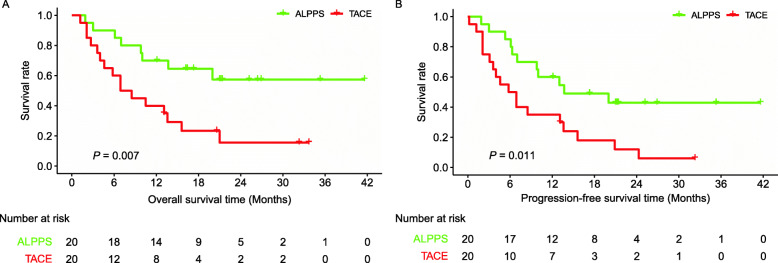
Survival analyses between ALPPS and the matched TACE groups. **a** The OS (*P* = 0.007) and **b** the PFS (*P* = 0.011) rates of patients in the ALPPS group were significantly better than those in the TACE group. Abbreviations: ALPPS, association liver partition and portal vein ligation for staged hepatectomy; TACE, transcatheter arterial chemoembolization; OS, overall survival; PFS, progression-free survival

Of the 66 patients with solitary huge HCC who underwent TACE, 20 were matched with the ALPPS group by using the PSM analysis (Table S3). TACE group had 1- and 3-year OS rates of 40.0% and 15.6%, respectively (Fig. 5a, *P* = 0.007). ALPPS group had 1- and the 3-year PFS rates of 35.0% and 6.0%, respectively (Fig. 5b, *P* = 0.011). Survival curves showed that the ALPPS group had significantly better outcomes than the TACE group.

## Discussion

Since its first public report in 2012 [9], ALPPS has been gradually promoted and applied in clinical practice. Increasing evidence suggests that ALPPS can remarkably improve the resectability of liver cancer and give patients with unresectable huge HCC an opportunity of curative resection [11, 20–22]. The present study has focused on patients with solitary huge HCC and compared the efficacy and the safety of ALPPS, one-stage resection, and TACE. Our results indicate that the OS of the ALPPS group is similar to that of the propensity score-matched one-stage resection group and significantly better than that of the matched TACE group.

One-stage resection and TACE are common clinical strategies for HCC treatment. Previous studies have compared the efficacy of one-stage resection and TACE in the treatment of solitary huge HCC, and the results show that one-stage resection can achieve improved outcomes [23, 24]. However, the feasibility of ALPPS, as a novel procedure of liver surgery, in solitary huge HCC has not been independently reported. The present study complements previous reports by adding ALPPS treatment to the analysis. Notably, our pivotal results show that ALPPS is safe and effective in unresectable cases with solitary huge HCC, comparable with one-stage resection, and superior to TACE on the curative effect.

The inadequate FLR is a direct challenge to the surgical treatment of solitary huge HCC especially one-stage resection, which is inclined to cause serious complications (such as liver failure and small-for-size syndrome after hepatectomy). By contrast, ALPPS can induce accelerated hypertrophy to acquire a sufficient volume of FLR through staged hepatectomy. Therefore, the appearance of ALPPS broadens the extent of indications for solitary huge HCC. In some centers, the conventional two-staged resection with portal vein ligation (PVL) or embolization (PVE) is also performed to induce the FLR hypertrophy in patients with unresectable HCC, but a relatively long waiting time is required to achieve adequate FLR volume compared with ALPPS [25, 26]. Approximately 20–38% of patients are ineligible for the curative resection after two-staged resection with PVL or PVE due to local tumor progression or extrahepatic metastasis [20, 27–29]. A recent study reports that ALPPS can improve the survival in patients with colorectal liver metastases and FLR < 30% compared with the conventional two-staged resection [30].

ALPPS is labeled as a high-risk operation due to its initially reported high morbidity and mortality [31]. However, a learning curve in ALPPS is observed, and things have gone into gradually reverse as improvement of patient selection, interstage management, and ALPPS technique [32, 33]. A study of the International ALPPS Registry shows that the 90-day mortality decreases from 17 to 4% in 2015 among 437 patients from 16 centers [13]. In this study, the 90-day mortality rate in 20 patients after ALPPS for solitary huge HCC is 5%. Our results are similar to the data reported in this longitudinal study. Noticeably, the decrease of ALPPS-associated risk is largely due to strict patient selection. From the data of the international registry, patients more than 60 years old have more severe postoperative complications (Clavien-Dindo IIIb or above) and higher mortality. Thus, elderly patients are poor candidates for ALPPS [34]. In addition, ALPPS are commonly used for patients with lower FLR volume; however, there is no consensus on the ideal index of FLR for ALPPS. A recent prospective trial suggested that ALPPS was performed on patients with FLR/SLV less than 30% when ALPPS was compared with conventional hepatectomy [20]. In clinical practice, potential liver diseases such as Child-Pugh grade, MELD score, fibrosis/cirrhosis, portal hypertension, and cholestasis are also important factors in surgical decision-making. Liver fibrosis/cirrhosis is a special concern for ALPPS in the treatment of HCC. A previous study demonstrated that the increase of FLR volume was negatively associated with the severity of liver fibrosis/cirrhosis [11]. Therefore, ALPPS should be carefully applied to patients with liver cirrhosis. For the perioperative management of ALPPS, the key is in the ALPPS-I stage. To assess the patient’s condition, except for monitoring conventional biochemical indicators, the pathological staging of liver cirrhosis was acquired based on the Ishak score [35], the classification of postoperative liver failure was evaluated according to the International Liver Surgery Research Group (ISGLS) [36], and the severity of postoperative complication was identified by the Clavien-Dindo classification [37]. CT imaging was performed on the 3rd, 7th, and 14th day after ALPPS-I operation, and the IQQA digital analysis software was used to calculate the FLR volume, which can assist in a more exact assessment of FLR increase to implement ALPPS-II operation as soon as possible. In recent years, a variety of modified ALPPS procedures was reported to improve the safety and the feasibility of ALPPS [38–42], greatly promoting the development and maturity of ALPPS technology. Moreover, some emerging liver image analysis systems also assist the improvement of ALPPS. For instance, preoperative FLR prediction using IQQA can be more effective and exact for patient selection [43]. Using the Liver Imaging Reporting and Data System (LI-RADS) to diagnose and classify HCC can achieve better screening and management [44, 45].

In addition, our results suggest that ALPPS can accelerate the FLR hypertrophy to reach sufficient volume for hepatic resection. However, severe liver fibrosis or cirrhosis may exert an adverse influence on the FLR hypertrophy between the ALPPS-I and the ALPPS-II stages. Patients with Ishak fibrosis scores of 5&6 have the lowest KGR compared with those with Ishak fibrosis scores of 1–4. Wang et al. [11] published their results with ALPPS that the median KGR of HCC patients with the absence of fibrosis/cirrhosis, mild fibrosis, moderate fibrosis, severe fibrosis, cirrhosis were 50.1, 19.0, 16.8, 19.8, and 9.6 cm^3^/day, respectively. Chan et al. [25] reported that the median KGR of patients with chronic hepatitis and cirrhosis were 24.6 and 20.7 cm^3^/day, respectively. Our results are approximate to these previous reports. Therefore, ALPPS should be prudently used in patients with cirrhosis or severe fibrosis.

The present study has identified the MELD score and the postoperative complications of ALPPS-II stage ≥ III as independent predictors of poor outcomes. In previous studies, a high MELD score and the Clavien-Dindo classification are demonstrated to be the risk factors for the poor prognosis of HCC [46–48]. Our data show that TACE is less effective in the treatment of solitary huge HCC. In most cases, TACE alone is not the optimal treatment for solitary huge HCC [7, 23, 24].

In this study, we screened the special type of HCC—solitary huge HCC—and demonstrated and compared the efficacy of ALPPS, one-stage resection, and TACE. However, several limitations need to be mentioned. First, the number of included cases in this single-center study is few, which is prone to potential bias. Second, the analysis of the modified ALPPS procedure and the two-staged resection with PVL or PVE is lacking because of the absence of these cases. Finally, the PSM analysis does not eliminate the selection bias completely. Thus, a rigorous multicenter randomized controlled trial should be designed to further verify the results.

## Conclusion

In conclusion, this work suggests that ALPPS is feasible for patients with solitary huge HCC. ALPPS enables the possibility of the curative resection of solitary huge HCC in patients who are perceived to have unresectable tumors in indications of conventional hepatectomy. The long-term OS after ALPPS is significantly superior to that after TACE and comparable with that after one-stage hepatic resection. For patients with unresectable solitary huge HCC, ALPPS is an alternative treatment that cannot be ignored.

## Supplementary Information

**Additional file 1: Table S1**. Univariable and Multivariable Cox regression analysis of risk factors for survival outcomes after ALPPS procedure.

**Additional file 2: Table S2**. Comparisons of baseline characteristics of patients with solitary huge HCC underwent ALPPS or one-stage resection before and after PSM.

**Additional file 3: Table S3**. Comparisons of baseline characteristics of patients with solitary huge HCC underwent ALPPS or TACE before and after PSM.

## Data Availability

The datasets analyzed during the current study are available from the corresponding authors on reasonable request.

## Abbreviations

ALPPS: Association liver partition and portal vein ligation for staged hepatectomy
HCC: Hepatocellular carcinoma
TACE: Transcatheter arterial chemoembolization
PSM: Propensity score matching
OS: Overall survival
PFS: Progression-free survival
FLR: Future liver remnant
TE: Transient elastography
CT: Computed tomographic
MRI: Magnetic resonance imaging
SLV: Standard liver volume
AFP: Alpha-fetoprotein
IQQA: Intelligent/interactive qualitative and quantitative analyses
MELD: Model for end-stage liver disease
KGR: Kinetic growth rate
PVL: Portal vein ligation
PVE: Portal vein embolization
