# Supplementary material for: Efficacy of the association liver partition and portal vein ligation for staged hepatectomy for the treatment of solitary huge hepatocellular carcinoma: a retrospective single-center study
Source: World J Surg Oncol. 2021 Mar 30;19:95. doi: 10.1186/s12957-021-02199-1 (PMC8011225; doi:10.1186/s12957-021-02199-1)
Supplement: Supplementary file 1 — Additional file 1: Table S1. Univariable and Multivariable Cox regression analysis of risk factors for survival outcomes after ALPPS procedure. [file 12957_2021_2199_MOESM1_ESM.docx]

Table S1. Univariable and Multivariable Cox regression analysis of risk factors for survival outcomes after ALPPS procedure

| Variable | Univariable Analysis | | Multivariable Analysis | |
| --- | --- | --- | --- | --- |
|  | HR (95% CI) | *P* | HR (95% CI) | *P* |
| Age, year | 0.972 (0.894~1.058) | 0.511 |  |  |
| Gender, female/male | 1.506 (0.185~12.275) | 0.702 |  |  |
| BMI, kg/m^2^ | 0.831 (0.625~1.104) | 0.202 | 0.765 (0.570~1.027) | 0.075 |
| AFP, ≥400 /<400ng/mL | 7.575 (0.916~62.637) | 0.060 | 13.351 (0.974~182.978) | 0.052 |
| Charlson comorbidity index | 1.106 (0.599~2.042) | 0.746 |  |  |
| MELD score | 1.483 (1.033~2.128) | **0.033** | 1.694 (1.035~2.771) | 0.036 |
| Child-Pugh class, A/B/C | 1.277 (0.438~3.725) | 0.655 |  |  |
| BCLC staging, A/B/C | 1.422 (0.623~3.247) | 0.403 |  |  |
| FLR/SLV before ALPPS, % | 1.030 (0.931~1.139) | 0.570 |  |  |
| Tumor size, cm | 1.071 (0.864~1.327) | 0.531 |  |  |
| ISGLS after ALPPS-I | 1.553 (0.367~6.572) | 0.550 |  |  |
| Clavien-Dindo after ALPPS-I,  classification ≥ III/< III | 2.890 (0.684~12.204) | 0.149 |  |  |
| ISGLS after ALPPS-II | 7.354 (0.512~105.659) | 0.142 |  |  |
| Clavien-Dindo after ALPPS-II,  classification ≥ III/< III | 6.077 (1.477~25.013) | **0.012** | 8.342 (1.270~54.777) | 0.027 |
| Ishak fibrosis score | 1.201 (0.743~1.942) | 0.454 |  |  |
| Macrovascular invasion, Y/N | 2.464 (0.494~12.283) | 0.271 | 6.099 (0.554~67.110) | 0.139 |

Abbreviations: ALPPS, association liver partition and portal vein ligation for staged hepatectomy; BMI, body mass index; AFP, alpha-fetoprotein; MELD, model for end-stage liver disease; BCLC, Barcelona Clinic Liver Cancer; FLR, future liver remnant; SLV, standard liver volume; ISGLS, International Study Group of Liver Surgery.
