# Supplementary material for: Efficacy of the association liver partition and portal vein ligation for staged hepatectomy for the treatment of solitary huge hepatocellular carcinoma: a retrospective single-center study
Source: World J Surg Oncol. 2021 Mar 30;19:95. doi: 10.1186/s12957-021-02199-1 (PMC8011225; doi:10.1186/s12957-021-02199-1)
Supplement: Supplementary file 2 — Additional file 2: Table S2. Comparisons of baseline characteristics of patients with solitary huge HCC underwent ALPPS or one-stage resection before and after PSM. [file 12957_2021_2199_MOESM2_ESM.docx]

Table S2. Comparisons of baseline characteristics of patients with solitary huge HCC underwent ALPPS or one-stage resection before and after PSM

|  | Before PSM | | | After PSM | | |
| --- | --- | --- | --- | --- | --- | --- |
|  | ALPPS  (n=20) | One-stage resection (n=110) | *P* value | ALPPS  (n=20) | One-stage resection (n=20) | *P* value |
| Age (years) | 47 (32~75) | 50 (17~75) | 0.478 | 47 (32~75) | 48 (31~72) | 0.825 |
| Gender, n (%)  female  male | 3 (15.0%)  17 (85.0%) | 22 (20.0%)  88 (80.0%) | 0.602 | 3 (15.0%)  17 (85.0%) | 4 (20.0%)  16 (80.0%) | 0.667 |
| BMI | 21.3 (18.0~30.1) | 22.5 (15.4~30.5) | 0.504 | 21.3 (18.0~30.1) | 21.4 (17.9~27.9) | 0.904 |
| Charlson comorbidity index | 4 (3~7) | 4 (2~16) | 0.687 | 4 (3~7) | 4 (3~9) | 0.896 |
| ECOG score  0  1  2 | 4 (20.0%)  13 (65.0%)  3 (15.0%) | 37 (33.6%)  52 (47.3%)  21 (19.1%) | 0.330 | 4 (20.0%)  13 (65.0%)  3 (15.0%) | 5 (25.0%)  12 (60.0%)  3 (15.0%) | 0.927 |
| AFP, n (%)  ≥400ng/mL  <400ng/mL | 12 (60.0%)  8 (40.0%) | 58 (52.7%)  52 (47.3%) | 0.548 | 12 (60.0%)  8 (40.0%) | 13 (65.0%)  7 (35.0%) | 0.744 |
| MELD score | 5 (2~11) | 5 (1~12) | 0.767 | 5 (2~11) | 5 (2~9) | 0.662 |
| Child-Pugh class  A  B | 19 (95.0%)  1 (5.0%) | 103 (93.6%)  7 (6.4%) | 0.815 | 19 (95.0%)  1 (5.0%) | 19 (95.0%)  1 (5.0%) | 1.000 |
| Tumor size (cm) | 14.5 (10.0~20.5) | 12.0 (10.0~21.5) | 0.029 | 14.5 (10.0~20.5) | 14.0 (10.0~18.5) | 0.836 |
| Macrovascular invasion, n (%)  Yes  No | 11 (55.0%)  9 (45.0%) | 59 (53.6%)  51 (46.4%) | 0.910 | 11 (55.0%)  9 (45.0%) | 11 (55.0%)  9 (45.0%) | 1.000 |
| Extrahepatic metastases, n (%)  Yes  No | 0 (0%)  20 (100%) | 0 (0%)  110 (100%) | 1.000 | 0 (0%)  20 (100%) | 0 (0%)  20 (100%) | 1.000 |

Abbreviations: HCC: hepatocellular carcinoma; ALPPS, association liver partition and portal vein ligation for staged hepatectomy; PSM, propensity score matching; BMI, body mass index, ECOG, Eastern Cooperative Oncology Group; AFP, alpha-fetoprotein; MELD, model for end-stage liver disease.
