# Supplementary material for: Efficacy of the association liver partition and portal vein ligation for staged hepatectomy for the treatment of solitary huge hepatocellular carcinoma: a retrospective single-center study
Source: World J Surg Oncol. 2021 Mar 30;19:95. doi: 10.1186/s12957-021-02199-1 (PMC8011225; doi:10.1186/s12957-021-02199-1)
Supplement: Supplementary file 3 — Additional file 3: Table S3. Comparisons of baseline characteristics of patients with solitary huge HCC underwent ALPPS or TACE before and after PSM. [file 12957_2021_2199_MOESM3_ESM.docx]

Table S3. Comparisons of baseline characteristics of patients with solitary huge HCC underwent ALPPS or TACE before and after PSM

|  | Before PSM | | | After PSM (caliper value=0.1) | | |
| --- | --- | --- | --- | --- | --- | --- |
|  | ALPPS (n=20) | TACE (n=66) | *P* value | ALPPS (n=20) | TACE (n=20) | *P* value |
| Age (years) | 47 (32~75) | 50 (17~77) | 0.569 | 47 (32~75) | 49 (26~73) | 0.327 |
| Gender, n (%)  female  male | 3 (15.0%)  17 (85.0%) | 8 (12.1%)  58 (87.9%) | 0.736 | 3 (15.0%)  17 (85.0%) | 1 (5.0%)  19 (95.0%) | 0.292 |
| BMI | 21.3 (18.0~30.1) | 22.3 (17.3~36.8) | 0.418 | 21.3 (18.0~30.1) | 21.5 (17.6~36.8) | 0.397 |
| Charlson comorbidity index | 4 (3~7) | 4 (2~10) | 0.597 | 4 (3~7) | 4 (3~8) | 0.288 |
| ECOG score, n (%)  0  1  2 | 4 (20.0%)  13 (65.0%)  3 (15.0%) | 27 (40.9%)  36 (54.60%)  3 (4.5%) | 0.102 | 4 (20.0%)  13 (65.0%)  3 (15.0%) | 6 (30.0%)  12 (60.0%)  2 (10.0%) | 0.726 |
| AFP, n (%)  ≥400ng/mL  <400ng/mL | 12 (60.0%)  8 (40.0%) | 34 (51.5%)  32 (48.5%) | 0.505 | 12 (60.0%)  8 (40.0%) | 12 (60.0%)  8 (40.0%) | 1.000 |
| MELD score | 5 (2~11) | 6 (1~23) | 0.036 | 5 (2~11) | 6 (2~10) | 0.114 |
| Child-Pugh class, n (%)  A  B  C | 19 (95.0%)  1 (5.0%)  0 (0%) | 54 (81.8%)  11 (16.7%)  1 (1.5%) | 0.815 | 19 (95.0%)  1 (5.0%)  0 (0%) | 19 (95.0%)  1 (5.0%)  0 (0%) | 1.000 |
| Tumor size (cm) | 14.5 (10.0~20.5) | 14.0 (10.0~25.0) | 0.599 | 14.5 (10.0~20.5) | 14.9 (10.2~25.0) | 0.365 |
| Macrovascular invasion, n (%)  Yes  No | 11 (55.0%)  9 (45.0%) | 34 (51.5%)  32 (48.5%) | 0.785 | 11 (55.0%)  9 (45.0%) | 11 (55.0%)  9 (45.0%) | 1.000 |
| Extrahepatic metastases, n (%)  Yes  No | 0 (0%)  20 (100%) | 5 (7.6%)  61 (92.4%) | 0.586 | 0 (0%)  20 (100%) | 0 (0%)  20 (100%) | 1.000 |

Abbreviations: HCC: hepatocellular carcinoma; ALPPS, association liver partition and portal vein ligation for staged hepatectomy; TACE: transcatheter arterial chemoembolization; PSM, propensity score matching; BMI, body mass index, ECOG, Eastern Cooperative Oncology Group; AFP, alpha-fetoprotein; MELD, model for end-stage liver disease.
